# Oro-dental manifestations of eating disorders: a systematic review

**DOI:** 10.1186/s40337-024-01050-8

**Published:** 2024-06-24

**Authors:** Leoluca Valeriani, Francesco Frigerio, Claudia Piciocchi, Gabriela Piana, Marco Montevecchi, Lorenzo Maria Donini, Edoardo Mocini

**Affiliations:** 1 Department of Biomedical and Neuromotor Sciences, University of Bologna, Bologna, 40125 IT Italy; 2 Department of Experimental Medicine, Sapienza University, Rome, 00185 IT Italy

**Keywords:** Anorexia nervosa, Bulimia nervosa, Oral health, Tooth erosion, Dental caries, Saliva, EDNOS

## Abstract

**Background:**

Eating disorders (EDs) pose a significant risk to health, especially when not diagnosed early. For several years EDs and oral health has been extensively studied, and now it is quite clear the existence of a correlation between specific oral manifestations and these disorders. While these oral signs could potentially aid early diagnosis of EDs, their identification and the eventual establishment of a correlation is currently heavily limited to the clinician’s experience. The present systematic review critically examines existing literature, offering an updated overview of oro-dental manifestations associated with EDs.

**Method:**

MEDLINE (via PubMed), Web of Science, Scopus, and grey literature were searched, and relevant epidemiological comparative studies were screened using the Rayyan software. No limitations have been imposed on the research regarding oro-dental outcomes, encompassing all medically diagnosed EDs. The quality of the studies was valuated using AXIS appraisal tool for cross-sectional studies.

**Result:**

Out of 3990 studies, 32 fulfilled the eligibility criteria and were included in the synthesis. The identified eating disorders include Anorexia Nervosa, Bulimia Nervosa and/or Eating Disorders Not Otherwise Specified, predominantly among female subjects, primarily originating from Europe. The evaluated oro-dental outcomes include dental erosion, caries, saliva assessment, hygiene-periodontal parameters, and mucosal tissue appearance. The association with erosion is confirmed while gingival recession, dentinal hypersensitivity, salivary flow thresholds and aspects relating to oral pathology are receiving increasing support from emerging evidence.

**Discussion:**

This trend emphasizes the critical role of the complete intraoral examination to detect significant oro-dental signs that may indicate the onset of an ED.

**Supplementary Information:**

The online version contains supplementary material available at 10.1186/s40337-024-01050-8.

## Abstract (plain language summary)

The article is a review of existing studies that explores the link between eating disorders and oral health issues. It found that people with eating disorders, including anorexia nervosa and bulimia nervosa, may experience dental problems such as tooth erosion, cavities, and altered saliva production. The review findings emphasize the importance of dental care providers recognizing these signs early and suggests better training for dental professionals. By doing so, they can help diagnose eating disorders sooner and recommend appropriate treatment. This approach aims to improve patients’ overall wellbeing by addressing both the oral health issues and the underlying eating disorders, making it essential for patients and medical teams to be aware of the interconnectedness between oral health and eating disorders.

**Supplementary Information:**

The online version contains supplementary material available at 10.1186/s40337-024-01050-8.

## Background

Feeding and eating disorders (EDs) are characterized by an enduring disturbance in eating habits, markedly impacting either an individual’s physical well-being and/or their psychosocial functioning, representing complex and multifaceted psychiatric conditions [1].

These disorders encompass various conditions, such as anorexia nervosa (AN), bulimia nervosa (BN), avoidant/restrictive food intake disorder, binge eating disorder, pica, rumination disorder, other specified feeding or eating disorder and unspecified feeding or eating disorder. EDs lead to clinically significant compromises in physical health, psychological well-being, and social functioning, typically manifesting as alterations in the quantity, quality, or frequency of ingested food, often accompanied by concerns regarding body weight, shape, or size [1].

Individuals with EDs are at significant risk of morbidity and mortality, regardless of their weight status. This risk is linked to various factors such as malnutrition (leading to conditions such as cardiac diseases and deterioration in bone density), uncontrolled eating with overnutrition (leading to obesity, diabetes, and metabolic syndrome), the use of compensatory mechanisms (which can cause electrolyte imbalances, resulting in a range of cardiovascular and neurological issues), and mood disturbances (potentially leading to suicidal tendencies) [2, 3].

The multifactorial etiology, which likely involves a combination of sociocultural, neurobiological, genetic, psychological, and interpersonal factors, makes it highly complex to determine causality. Moreover, the time lapse between onset (which can often be unclear) and the identification of these disorders spans several years. Individuals with eating disorders face physical complications, psychological comorbidities, reduced quality of life, relational challenges, emotional distress, social isolation, and economic disadvantage, often occurring alongside coexisting mood disorders and substance abuse [4, 5].

An early diagnosis and intervention are crucial to minimize the risk of serious medical and psychological complications, as well as to prevent the chronicization of the disorder. However, the difficulty in recognizing risk factors and the often limited presence of physical symptoms in the early stages of onset makes EDs challenging to detect within primary care settings [6]. Indeed, eating disorders, which frequently originate in adolescence with low rates of spontaneous remission, remain undiagnosed and undetected by healthcare professionals until adulthood [7]. In this context, the association between oral health and eating behaviors may hold significant importance, as it could enable early and reliable screening.

To the best of our knowledge, the latest two systematic reviews (2014 and 2015) concur in identifying specific oral manifestations that are more prevalent in patients with EDs [8, 9]. Hermont et al. found a significant association with dental erosion, while Kisely et al., in addition to dental erosion, observed higher DMFS (Decayed, Missing, Filled Surfaces) scores and reduced salivary flow. Both reviews underscored the need for further studies in this area, encompassing a broader range of oro-dental outcomes.

Currently, the diagnosis of dental lesions associated with eating disorders, crucial for potential early screening, depends on dentists’ clinical experience or the identification of particularly overt signs, primarily dental erosion. However, overt signs might indicate an ongoing disease that has been present for a while, suggesting a failure in achieving an early diagnosis. To date, the scientific literature lacks a comprehensive analysis or review that consolidates all potential clinical manifestations. These aspects form the rationale for the current systematic literature review, aimed at assessing the oro-dental manifestations of feeding and eating disorders.

## Methods

This systematic literature review was conducted following the Preferred Reporting Items for Systematic Reviews and Meta-Analyses (PRISMA) guidelines [10].

### Search strategy

A systematic electronic search (finalized in September 2023) was performed in three different databases (MEDLINE via PubMed, Scopus, and Web of Science) to detect pertinent studies.

The following terms were searched: (“anorexia” OR “bulimia” OR “binge eating” OR “eating disorder*” OR “appetite disorder*” OR “binge-eating” OR “hyperphagia”) AND (“oral health” OR “oral hygiene” OR “tooth*” OR “dent*” OR “temporomandibular” OR “mouth” OR “oral status” OR “oral manifestation*” OR “oral cavity” OR “oral mucosa*” OR “saliva*”). Additional file details the search string used in each database.

### Study selection

Only comparative studies examining the association between eating disorders and one or more oral related aspects were considered. All studies encompassing any oral or dental outcomes were included.

All eating disorders were taken into consideration.

Human studies involving participants with a physician-confirmed diagnosis of EDs were included, while studies of people with severe mental illnesses, primary alcohol or substance use disorders, intellectual disability, and other psychological disorders that could impact oral health were excluded.

Reports, case, reviews, meta-analysis, book chapters, expert opinions and conference abstracts were excluded, but there was no restriction regarding epidemiological study design. No limit on publication year was imposed, but only articles published in English were considered eligible.

A three-step procedure was applied (titles, abstracts, and full texts were screened in sequence) after excluding duplicates from the search results. The references of included studies were also checked to identify other potentially relevant studies. Two researchers conducted the search process autonomously (V.L. and M.E.); disagreements were solved by discussion and the mediation of a third reviewer (M.M.).

### Data extraction

General article information (first authors, year of publication), study characteristics (study design, country of origin, sample size determination, site of recruitment for individuals with ED), and participant traits (age, gender, ED diagnosis and applied diagnostic criteria) were independently extracted by two reviewers (V.L. and M.E.). Moreover, information on the eating disorder and outcomes used in the respective studies and the main results were systematically synthesized and analysed.

### Quality assessment

The quality assessment of the reviewed studies was conducted by two reviewers (V.L. and M.E.) using the Appraisal tool for Cross-Sectional Studies (AXIS) [11].

## Results

### Study selection

The systematic search returned 3990 references (1581 in Scopus, 1241 in Web of Science, 1168 in MEDLINE via PubMed). After removing duplicates, 2015 studies were eligible for title screening. Following the screening of titles and abstracts, 1939 articles were excluded. Full-text examination was then conducted on 76 articles, and finally 32 papers were included in this review. The references of included studies were also checked but it did not yield the identification of further studies that met our eligibility criteria. Additional Fig. 1 presents a flowchart depicting the screening and selection processes. The detailed PRISMA checklist can be found in Additional Materials Table 1.

**Table 1:** Participant characteristics in the studies included in the systematic review

Author	Nation	Study design	Number of Participants	Matching	Eating Disorder	Mean age, y	Gender	ED Recruitment Location	Diagnosis	Sample Size Determination	
Aframian et al. 2010 [18]	Israel	Cross sectional	22 BN 26 C	No	BN	BN: 27.7 ± 10.6 C: 52.1 ± 9.1	BN: All female C: 11 M, 25 F	NR	DSM-IV	Yes	
Altshuler et al. 1990 [22]	USA	Cross sectional	40 BN 40 C	Yes	BN	ED: 23.9 ± 5.5 C: 24.9 ± 6.1	All female	Eating Disorder Unit/ Outpatients	DSM-III-R	No	
Blazer et al. 2008 [23]	Israel	Cross sectional	26 BN 26 C	Yes	BN	BN: 24 ± 7 C: NR	All female	Eating Disorders Clinic	DSM-IV	No	
Chiba et al. 2019 [39]	Brazil	Cross sectional	30 AN and BN 30 C	No	MIX	ED: 31.1 ± 12.7 C: 28.9 ± 9.7	NR	Specialized Mental Health Clinic	NR	No	
Chiba et al. 2022 [40]	Brazil	Cross sectional	30 AN and BN 30 C	No	MIX	ED: 31.1 ± 12.7 C: 28.9 ± 9.7	All female	Specialized Mental Health Clinic	NR	Yes	
Dynesen et al. 2008 [31]	Denmark	Cross sectional	20 BN 20 C	Yes	BN	BN: 23.8 ± 4 C: 23.1 ± 2	All female	Psychiatric practice and psychologic research project and among students	DSM-IV	No	
Garrido-Martínez et al. 2019 [43]	Spain	Cross sectional	59 EDNOS 120 C	Yes	MIX	Whole sample: 27.62 (19–44)	ED: All female C: NR	Attending the hospital’s Clinical Nutrition Unit	DSM-V	No	
Johansson et al. 2010 [29]	Sweden	Cross sectional	32 EDNOS 14 AN 8 BN 54 C	Yes	MIX	ED: 21.5 (10–50) C: NR	ED: 50 F, 4 M C: 50 F, 4 M	Eating Disorder Clinic/ Outpatient	NR	No	
Johansson et al. 2012 [33]	Sweden	Cross sectional	32 EDNOS 14 AN 8 BN 54 C	Yes	MIX	ED: 21.5 (10–50) C: NR	ED: 50 F, 4 M C: 50 F, 4 M	Eating Disorder Clinic/ Outpatients	NR	No	
Johansson et al. 2015 [34]	Sweden	Cross sectional	32 EDNOS 14 AN 8 BN 54 C	Yes	MIX	Whole sample: 21.5 ± 6.8 (10–50)	ED: 50 F, 4 M C: 50 F, 4 M	Eating Disorder Clinic/ out-patient	NR	No	
Jones and Cleaton-Jones 1989 [12]	South Africa	Cross sectional	11 BN 22 C	Yes	BN	BN: 29.8 ± 8.4 C: 28.9 ± 9.0	All female	Private dental office	NR	No	
Lesar et al. 2022 [30]	Croatia	Cross sectional	27 AN 6 BN 17 EDNOS 51 C	No	MIX	Whole sample: 14.34 ± 1.99	All female	Hospitalized in the Department of Eating Disorders of the Pediatric Clinic	DSM-V	No	
Lourenço et al. 2018 [32]	Portugal	Cross sectional	18 AN 15 BN 33 C	No	MIX	ED: 28.2 ± 10.1 C: 23.2 ± 3.3	All female	Hospitalized outpatients	DMS-V	No	
Manevski et al. 2020 [35]	Serbia	Cross sectional	30 Purging BN 30 C	Yes	BN	BN 24.6 ± 4.42 C: NR	ED: 28 F, 2 M C: 28 F, 2 M	Psychiatric Clinic	NR	No	
Mascitti et al. 2019 [36]	Italy	Cross sectional	25 AN 25 C	Yes	AN	AN: 24.5 ± 9.2 C: 24.2 ± 5.4	All female	Undergoing psychiatric and/or medical outpatient treatment	DSM-IV	No	
Milosevic and Dawson 1996 [15]	United Kingdom	Cross sectional	19 BN (9 TW+, 10 TW-) 10 C	No	BN	BN TW+: 28.6 BN TW-: 27.3 C: 33.2	ED: 18 F, 1 M	NR	NR	No	
Ohrn et al. 1999 [41]	Sweden	Cross sectional	46 BN 25 EDNOS 7 MIXED AN and BN 3 AN 52 C	No	MIX	ED: (17–47) median 25 C: (19–41) median 24.	ED: 79 F, 2 M C: 48 F, 4 M	Day care psychiatric clinic/ Outpatient	DSM-III-R	No	
Pallier et al. 2019 [42]	France	Cross sectional	36 AN 34 BN 70 C	No	MIX	ED: 32.1 ± 9.1 C: 30.2 ± 4.7	All female	Referred to the department of Psychiatry and Addiction	NR	Yes	
Panico et al. 2018 [16]	Argentina	Cross sectional	46 BN 13 EDNOS 6 AN (3 Restrictive and 3 Purgative) 65 C	Yes	MIX	ED: 21.6 (12–32) C: 23.21 (14–31).	All female	Anorexia and Bulimia Fight Association Institute	DSM-IV	No	
Paszynska et al. 2006 [24]	Poland	Cross sectional	33 BN 51 C	Yes	BN	BN: 21.2 ± 3.2 C: 25.5 ± 4.6	All female	Referred to the University’s Clinical Psychiatric Department.	DSM-IV	No	
Paszynska et al. 2013 [19]	Poland	Cross sectional	33 vomiting BN on fluoxetine 51 C	Yes	BN	BN: 21.2 ± 3.2 C: 25.5 ± 4.6	All female	NR	ICD 10 (code F 50.2) DSM-IV criteria (code 307.51)	No	
Paszynska et al. 2014 [25]	Poland	Cross sectional	31 AN 40 C	Yes	AN	AN: 15 ± 2 C: 14 ± 1	All female	NR	ICD 10 (code F 50.0) DSM -IV (code 307.1)	No	
Paszynska et al. 2015 [26]	Poland	Cross sectional	28 AN 38 C	Yes	AN	AN: 15 ± 2 C 14 ± 1	All female	NR	ICD 10 (code F 50.0) DSM-IV (code 307.1)	No	
Paszynska et al. 2017 [27]	Poland	Cross sectional	20 AN 21 C	Yes	AN	AN: 15.5 ± 2.1 C: 16 ± 1.2	All female	NR	ICD 10 (code F 50.0) DSM–IV (code 307.1) DSM–V (code F 50.01)	No	
Paszynska et al. 2022 [13]	Poland	Cross sectional	117 AN 103 C	Yes	AN	AN: 14.9 ± 1.8 C: 15.0 ± 1.8	All female	Psychiatric Unit for Child and Adolescents. Hospitalized in acute phase	ICD-10 (code F50.1) DSM-5 (code 307.1)	No	
Pereira de Souza et al. 2018 [37]	Brazil	Cross sectional	26 AN (19 purging and 7 restricting) 16 BN 22 C	No	MIX	AN sR: 37 (20–47) AN sP: 33 (25–40) BN: 33 (24–41) C: 26 (22–30)	AN sR: All female AN sP: 17 F, 2 M BN: 13 F, 3 M C: 17 F, 5 M	Dental Clinic of the Institute of Psychiatry of the Hospital/ Outpatients	DSM-V ICD 10	No	
Philipp et al. 1991 [20]	Germany	Cross sectional	41 BN 11 AN 50 C	No	MIX	AN: 22 (18–27) BN: 25 (17–39) C: 27 (17–37)	All female	Outpatients of Institute of Psychiatry and inpatients of the Psychosomatic Hospital	DSM-III	No	
Riad et al. 1991 [17]	UK	Cross sectional	28 BN 30 C	No	BN	BN: 24.6 ± 5.9 C: 26.8 ± 6.3	ED: 26 F, 2 M C: NR	Hospital bulimia clinic	DSM-III	No	
Rytömaa et al. 1998 [14]	Finland	Cross sectional	35 BN 105 C	Yes	BN	BN: 25.3 ± 6.8 C: 25.7 ± 7.0	All female	Departments of Psychiatry and Adolescent Psychiatry/ Outpatients.	DSM-III-R	No	
Schlueter et al. 2012 [28]	Germany	Cross sectional	14 vomiting BN (7 with erosion, 7 without) 14 C without erosion	Yes	BN	Whole sample 27.1 ± 5.6	NR	NR	ICD-10 (code F50.1)	No	
Sirin et al. 2012 [38]	Turkey	Cross sectional	43 AN (19 binge/purge, 24 restrictive) 17 BN (purging) 12 EDNOS 72 C	Yes	MIX	Whole sample: 23.51 ± 7.3 (13.51)	All female	Referred by the Department of Psychiatry	DSM-IV-TR	No	
Touyz et al. 1993 [21]	Australia	Cross sectional	15 AN (all restricting) 15 BN 15 C	Yes	MIX	AN: 20.1 ± 8.3 BN: 19.1 ± 3.8 C: 22.1 ± 3.3	All female	Inpatients	DSM-III-R	No	

Legend: AN: Patients with Anorexia Nervosa; BN: Patients with Bulimia Nervosa; C: Control Group; ED: Eating Disorders, EDNOS: Eating Disorder Not Otherwise Specified; MIX: Multiple ED Diagnosis; DSM: Diagnostic and Statistical Manual of Mental Disorders; ICD: International Classification of Diseases; F: Females; M: Males; TW: Tooth Wear; sR: Restricting subtype; sP: Purging subtype; NR: Not Reported

### Data synthesis

The main characteristics of the 32 included studies (2732 participants, 1309 with eating disorders and 1423 healthy controls) are summarized in Table 1. Most studies includedonly female participants (*n* = 19), with a few studies including both sexes (*n* = 9), while the gender of participants in 4 studies was not reported. No studies exclusively examining male subjects were found.

Regarding the region of origin, the studies were sourced predominantly from Europe (*n* = 23), followed by 4 studies from South America, 2 from Asia, and 1 each from North America, Oceania, and Africa.

All studies are cross-sectional, and they were published between 1989 and 2022. The cohort size ranged from *n* = 11 [12] to *n* = 117 [13].

Out of the 32 studies, 5 examined individuals with anorexia nervosa, 12 exclusively focused on bulimia nervosa patients, while 8 studies assessed both anorexia nervosa and bulimia nervosa. Additionally, in 7 studies, the specific eating disorder being investigated was either not reported or included the EDNOS (Eating Disorder Not Otherwise Specified) group. None of these studies addressed binge eating disorders.

Regarding the oral factors measured, the most common were erosion (14 studies, 1396 patients), caries (14 studies, 1500 patients), salivary function (19 studies, 1502 patients), pH (11 studies, 811 patients), periodontal and hygienic parameter (11 studies, 1190 patients) and oral mucosal tissues (7 studies, 701 patients). The rest of the oral-related factors included: hypersensitivity (2 studies), temporomandibular disorders (2 studies), parafunctional habits (1 study) and malocclusion (1 study). Nearly all the studies measured oral outcomes through specialist clinical examinations, while only a few outcomes were derived from questionnaires (such as on dry mouth/xerostomia, temporomandibular disorders, or parafunctional habits).

### Quality of studies

An overview of the AXIS quality assessment of the included studies is displayed in additional file (Additional Table 2). The overall quality of studies was 11.25. The quality scores span a range from 7 to 17. Two studies had a quality score of 7 [14, 15], two scored 8 [16, 17], four scored 9 [18–21], seven scored 10 [22–28], two scored 11 [29, 30], three scored 12 [12, 31, 32], six scored 13 [33–38], four scored 14 [39–42], one scored 15 [43], and one scored 17 [13]. The majority of studies employed a suitable design to address their research inquiries and, with the exception of one, all studies had clear study aims. However, it is noteworthy that only one study accounted for non-responders in their analyses. The primary methodological deficiencies were predominantly associated with sample size limitations, unclear matching criteria, and study designs that were not clearly specified by the authors. Additionally, there was notable variability in the descriptions of patient characteristics across studies, incomplete reporting of results in some instances, and inconsistent definitions of outcome measures. Despite the range of quality scores, the overall quality of the included studies can be considered moderate. While they generally exhibit a suitable design for their research objectives, significant methodological limitations, such as small sample sizes, ambiguous matching criteria, and lack of clarity in study designs, somewhat undermine their robustness.

**Table 2:** Main results dental erosion

Author	Assessment criteria	Main results
Altshuler et al. 1990 [22]	Presence of erosion was noted when a loss of enamel on a tooth surface was accompanied by exposure of dentin and/or alteration of morphology.	78% of bulimic subjects demonstrated an average of 7.6 eroded tooth surfaces, which most frequently affected the lingual surfaces of the maxillary anterior teeth. Erosion was observed in one (2.5%) control subject. Mean eroded tooth surfaces ± SD (range). BN: 7.6 ± 7.6 (0–31) vs. C: 0.2 ± 0.8 (0–4) (*p* = 0.001). Vomit duration was related to the number of eroded tooth surfaces (*p* < 0.01). After 6 months, most BN patients exhibited one or more eroded tooth surfaces. All BN subjects exhibited erosion after 5 years.
Dynesen et al. 2008 [31]	Impressions in a silicone material. Photographs. Clinical inspection according to Larsen et al. with modifications [59]. Modification: additional score of 0.5 concerning the facial and oral crown surfaces when “original developmental structures, perichymata, are present on less than one-half of the surface”.	The dental erosion score was significantly higher in the BN group. Mean score (range). BN: 1.0 (0.4–2.8) vs. C: 0.6 (0.3–0.9) (*p* = 0.019).
Garrido-Martínez et al. 2019 [43]	Johansson et al., 1996 [60].	Erosion was significantly greater in the ED group than in the C (*p* < 0.001). The frequency of vomiting and dental erosion showed a statistically significant association. Mean (%). ED: 45 (76.3%) vs. C: 11 (9.2%) (*p* < 0.001).
Johansson et al. 2012 [33]	Grading of dental erosion was performed clinically using an ordinal scale on the maxillary incisors and canines [60]. The severity was expressed using the mean index value of all graded surfaces for each participant.	Severity of dental erosion was significantly higher in the ED group. Median (range). ED: 1.5 (0.5–2.7) vs. C: 0.7 (0.0–2.3) (*p* = 0.001). Severe erosion extending into dentin or close to dentinal exposure on large surfaces, on one tooth or more, was found in 36% of the ED group and in 11% of the control group (*p* = 0.005).
Jones and Cleaton-Jones 1989 [12]	Erosions were defined as “dished out” areas of enamel, or enamel and dentin, on the buccal or lingual tooth surface. They were graded by depth (absent, into enamel only, into enamel and dentin, into pulp) and by area (< 1/3; 1/3 − 2/3; >2/3 of the tooth surface).	Erosion was significantly higher in BN (69%) vs. C group (7%) on buccal/lingual surfaces (χ² = 7.01, *p* < 0.001). On the buccal surfaces, the bulimics’ erosions were mainly in the maxillary premolar and canine teeth, and in the premolar and molar mandibular teeth. On the palatal/lingual surfaces, erosions affected particularly the incisor and canine teeth in both jaws. In C group, erosions were limited to enamel without dentin involvement while in ED patients it extended to the dental pulp. In C group (differently to ED), erosions never affected the molars, and for premolars, they were limited to the buccal and lingual aspects.
Lourenço et al. 2018 [32]	Dental erosion was clinically diagnosed. A severity scale was used to score each tooth surface: 0 (without lesion), 1 (lesion limited to enamel), 2 (lesion affecting dentin), 3 (lesion affecting dental pulp), and 4 (missing or excluded).	Erosion was significantly higher in ED compared to C (*p* < 0.001). ED individuals with vomiting tendencies exhibited significantly greater erosion compared to those without (*p* < 0.001). There was no difference between the ED non-vomit group and C group (*p* = 0.16).
Manevski et al. 2020 [35]	Basic Erosive Wear Examination [44].	In the BN group, there were significant differences (*p* < 0.05) observed in comparison to C concerning the following: quantity of patients with erosion (27 (90%) vs. 19 (63.3%)), total count of erosions (82 vs. 54), and mean ± SD erosions number (2.73 ± 1.53 vs. 1.8 ± 1.69). Erosion distribution among tooth groups or arches didn’t differ significantly (*p* = 0.791), but oral surfaces were more affected in BN patients (43.9% vs. 20.4%), while vestibular surfaces were higher in the C group (22% BN vs. 44.4% C). Mean ± SD BEWE index score. BN: 2.67 ± 1.6 vs. C: 1.23 ± 1.19 (*p* < 0.05). Dental erosions were significantly more often present in purging bulimics compared to the controls (*p* < 0.05), with significantly higher total and average number (t = 2.243, *p* < 0.05) of erosion per patient.
Mascitti et al. 2019 [36]	Basic Erosive Wear Examination [44].	Among AN patients, 76% showed several dental erosions, with a mean BEWE score of 5.24 ± 4.47. A total of 104 dental erosions were found: 75% were classified as initial erosion, 24% as moderate and 1% as severe. The dental surfaces most frequently involved were the occlusal surfaces of the molar and premolar regions of the mandible. BEWE mean number ± SD. AN: 5.2 ± 4.5 vs. C: 0.6 ± 0.7 (*p* < 0.05).
Ohrn et al. 1999 [41]	Dental impressions. Photographs. Lussi et al. modification of Eccles system for Tooth Wear [61].	Tooth wear differed between ED and C in both grades 1 + 2 and grade 1 (*p* < 0.001).
Pallier et al. 2019 [42]	Basic Erosive Wear Examination [44].	Differences (*p* < 0.01) were noted between ED and C patients. C group consistently had BEWE index values ≤ 2. In contrast, AN showed ≤ 2 values in 58.3%, BN in 23.5%. BN patients had a ≥ 3 BEWE score in 76.5%, AN in 41.7%, and none in the control group (*p* < 0.01).
Paszynska et al. 2022 [13]	Basic Erosive Wear Examination [44].	A BEWE score ≤ 2 was detected in 18.9% of AN patients as compared with C (2.9%) (*p* < 0.001). Score ≥ 3 was observed in 0% of the C and in 17.9% of the AN group. Total n BEWE ≥ 1 = AN: 22 (18.9%) vs. C: 3 (2.9%) (*p* < 0.001). In the C group, all BEWE scores were ≤ 2, while in the AN group, only 1 BEWE score was ≤ 2, with the others ranging from 3 to 13. Total n BEWE ≥ 1 ± SD, median (range). AN non-purging (*n* = 99): 0.3 ± 1.3, 0 (0–8) vs. AN purging (*n* = 18): 5.6 ± 2.5, 5 (3–12) (*p* < 0.001).
Philipp et al. 1991 [20]	Number and degree of decalcified dental surfaces.	All patients with ED had significantly more enamel erosions than C persons. N enamel erosion, mean ± SD. AN: 6.8 ± 5.7 vs. BN: 6.5 ± 4.9 vs. C: 1.1 ± 2.0 (*p* < 0.001). BN patients had very severe erosions affecting particularly the palatinal aspects of the upper incisor, canine and premolar teeth.
Rytomaa et al. 1998 [14]	Dental erosion was classified as one of three grades on buccal, lingual, and occlusal/ incisal surfaces.	Tooth erosion, abrasion, and attrition were 1.5 ± 6 times more frequent among bulimics than controls (*p* < 0.05). N subjects with erosion. BN: 22/35 (63%) vs. C: 12/105 (11%) (*p* < 0.01).
Touyz et al. 1993 [21]	NR	Seven bulimic patients displayed erosion of tooth structure (6.1% of surfaces examined). This was significantly more than the anorexics (1.0% of surfaces). Both groups of patients had significantly more surfaces with erosion than the control subjects (no eroded surfaces) (*p* < 0.05).

Legend: AN: Patients with Anorexia Nervosa; BN: Patients with Bulimia Nervosa; C: Control Group; BEWE: Basic Erosive Wear Examination; ED: Patients with Eating Disorders; SD: Standard Deviation; BEWE: Basic Erosive Wear Examination

## Association between eating disorder and oral outcomes

### Dental erosion

Dental erosion was assessed across 14 studies, encompassing a total of 1,396 patients. Among these, 5 studies exclusively involved individuals with bulimia nervosa, 2 studies focused on anorexia nervosa, and 7 studies included a combination of various eating disorders. The primary findings of these studies are summarized in Table 2. Five studies employed methods previously established and documented in the literature, four utilized the BEWE (Basic Erosive Wear Examination) [44] method, four studies employed alternative methods, and one study did not report. All articles on this topic found an association between EDs and erosion.

### Dental caries

Dental caries was evaluated in 14 studies, involving a total of 1,500 patients. Out of these, 4 studies exclusively enrolled participants with bulimia nervosa, 2 studies concentrated on anorexia nervosa, and 8 studies encompassed a mix of different eating disorders. The primary findings of these studies are summarized in Table 3. Most studies (n = 10) utilized either DMFT or DMFS (Decayed, Missing, Filled Tooth or Surfaces), while a few employed DMF (n = 2). In some studies (n = 7), the individual components ‘decayed,’ ‘missing,’ and ‘filled’ were also assessed separately, or pre-cavitation lesions were evaluated, or the areas were categorized into approximal and bucco-lingual.

**Table 3:** Main results dental caries

Author		Assessment criteria	Main results	
Altshuler et al. 1990 [22]		DMFS [62].	No difference in mean DMFS score (*p* > 0.05). BN group averaged higher caries, with 78% having at least one lesion, mostly pit and fissure (65%). Smooth surface caries (35%) were also common with enamel decalcification. Missing and filled surfaces counts similar. BN mean ± SD (range) decayed surface count was 41 ± 4.0 (0–18), higher than C’s 1.5 ± 2.0 (0–8) (*p* = 0.001).	
Garrido-Martínez et al. 2019 [43]		The presence of caries was determined visually using the DMF index.	No statistical difference in DMF index. Mean DMF ± SD. ED: 6.75 ± 3.79 vs. C: 6.39 ± 3.41 (*p* > 0.05).	
Johansson et al. 2012 [33]		DMFS and DMFT. Dental radiographs for proximal caries. Dental caries was recorded following a clinical visual-tactile inspection.	No differences between groups. Median DMFT (range). ED: 5.0 (0–18) vs. C: 4.0 (0–16) (*p* > 0.05). Median DMFS (range). ED: 6.0 (0–46) vs. C: 6.0 (0–37) (*p* > 0.05).	
Jones and Cleaton-Jones 1989 [12]		World Health Organization (1977) caries diagnostic criteria were used, and radiolucent areas in enamel or dentin on bite-wing radiographs were recorded as caries. DMFS.	One subject in the C group (4,5%) and two in the BN group (18%) were caries-free. Mean DMFS ± SD (median). BN: 19.1 ± 15.9 (13.5) vs. C: 27.9 ± 22.6 (36) (*p* > 0.05).	
Lourenço et al. 2018 [32]		DMFS and DMFT. Tooth decay evaluation was conducted by visual and probe inspection.	Carious lesions were more prevalent in ED compared to C, with no difference observed between vomit and non-vomit ED patients. Mean DMFT ± SD. ED: 8.78 ± 7.0 vs. C: 4.12 ± 3.9 (*p* < 0.02). Mean DMFS ± SD. ED: 23.72 ± 31.8 vs. C: 5.55 ± 6.1 (*p* < 0.02). Mean active decay ± SD. ED: 0.78 ± 0.4 vs. C: 0.27 ± 0.4 (*p* < 0.001).	
Manevski et al. 2020 [35]		DMFT.	No difference in DMFT index (t = 0.741, *p* = 0.461). Average number of decayed (t=-0.917, *p* = 0.363), missing (t = 1.969, *p* = 0.054) and filled teeth (t = 0.787, *p* = 0.434) did not find significant differences between two groups.	
Mascitti et al. 2019 [36]		DMFT.	Mean DMFT for AN was 6.80 ± 3.76 (range 0–12) with 40 caries in 14 patients (mean value of 1.60 ± 2.08; range 0–7) and 3.04 ± 2.05 (range 0–7) missing teeth. 16 AN patients presented 54 dental fillings (mean value of 2.16 ± 1.99; range 0–6). Significant difference in DMFT score and in the Missing and Filled components, but not in the Decayed one. Mean DMFT ± SD. AN: 6.8 ± 3.8 vs. C: 4.3 ± 2.2 (*p* < 0.05).	
Ohrn et al. 1999 [41]		DMFS. Dental radiographs. Dental impressions. Photos.	No difference in decayed surfaces (DS), but a significant difference (*p* < 0.01) was observed in mean ± SD decayed and filled surfaces (ED: 13.5 ± 9.8 vs. C: 9.4 ± 8.6) and DMFS (ED: 15.3 ± 10.9 vs. C: 10.8 ± 9.1). The difference in mean ± SD DS was evident (*p* < 0.05) when considering the age interval 21–30 (ED: 2.0 ± 2.8 vs. C: 0.7 ± 1.1).	
Pallier et al. 2019 [42]		DMFT.	ED patients had a higher DMFT score than controls. Mean DMFT ± SD. ED: 7.9 ± 7.5 vs. C: 4.7 ± 4.8 (*p* < 0.01). Analyses within subgroups revealed that AN patients had a higher mean value compared to BN (8.2 ± 7.3 vs. 7.5 ± 7.8, respectively), but this difference was not statistically significant (*p* = 0.91).	
Paszynska et al. 2022 [13]		DMFT.	37.6% of AN vs. 11.7% of C were affected by dental caries. There was a significantly higher DMFT score than in the C (3.8 ± 4.5 vs. 1.9 ± 2.1, *p* < 0.005), as well as the number of decayed teeth (1.2 ± 2.6 vs. 0.1 ± 0.4, *p* < 0.001), the number of missing teeth (0.1 ± 0.5 vs. 0, *p* < 0.02). Mean decayed ± SD, median (range). AN: 1.2 ± 2.6, 0 (0–21) vs. C: 0.1 ± 0.4, 0 (0–2) (*p* < 0.001). Mean DMFT ± SD, median (range). AN: 3.8 ± 4.5, 2 (0–21) vs. C: 1.9 ± 2.1 2 (0–10) (*p* = 0.005).	
Philipp et al. 1991 [20]		DMF.	Caries incidence determined by the DMF-value was different in patients suffering from eating disorders and in healthy control persons. Mean DMF ± SD. AN: 11.3 ± 5.4 vs. BN: 14.2 ± 6.4 vs. C: 15.3 ± 4.2 (*p* < 0.009).	
Rytomaa et al. 1998 [14]		Bitewing radiographs. Dental caries was recorded on the basis of WHO criteria: for carious surfaces, cavitation (DS) and pre-cavitation grades (DSini) were recorded.	No significant differences in terms of DMFS and DS (*p* > 0.05). BN individuals exhibited greater and significant (*p* < 0.05) pre-cavitation caries (5.8 ± 4.6 vs. 3.5 ± 2.0), approximal caries lesions (DS + DSini proximal) (4.7 ± 4.4 vs. 2.8 ± 1.6), and buccal-lingual caries (DS + DSini bucco-lingual) (1.2 ± 1.5 vs. 0.6 ± 0.9).	
Sirin et al. 2012 [38]		Panoramic and periapical radiography. The tooth status was classified as one of the following: sound, missing, radiographically detectable untreated carious lesion, restored, restored and carious lesion, restored and root canal treatment, restored and carious lesion and root canal treatment, crown, crown with carious lesion, crown and root canal treatment, and crown and carious lesion and root canal treatment. A carious lesion was defined [63]. The periapical status was evaluated using the periapical index (PAI [64]).	The percentage of untreated carious lesions among unrestored teeth was found to be statistically higher in the ED group (15.9%) compared with C patients (10.50%) (χ²= 20.59, d.f. = 4; *p* < 0.05). Mean number of teeth with at least one radiographically detectable untreated carious lesion in the ED group (3.84 ± 2.78) was significantly higher than C group (2.69 ± 1.52) (*p* < 0.05). When restored and missing surfaces were excluded, 449 (12.8%) approximal surfaces of the ED group and 259 (7.03%) approximal surfaces of the C group were found to have a carious lesion (χ² = 68.53, d.f. = 4; *p* < 0.01). No significant differences were found in depths of carious lesions and distributions of the occlusal lesions (*p* > 0.05). ED patients had a significantly higher mean number of untreated caries lesions (1.12 ± 1.2) in the mandibular posterior region compared to C patients (0.7 ± 0.93) (*p* < 0.05). Percentage of teeth with periapical pathologies (PAI scores ≥ 3) was significantly greater in the ED compared to C group (4.82% vs. 2.96%) (χ²= 9, 52, d.f. = 4; *p* < 0.05). The mean number of teeth with PAI scores of ≥ 3 in the ED group (1.31 ± 1.27) was significantly higher than that of the C group (0.77 ± 0.84) (*p* < 0.05).	
Touyz et al. 1993 [21]		DMFT.	No statistically significant differences between groups. Mean DMFT ± SD. AN: 4.4 ± 4.7 vs. BN: 3.9 ± 2.9 vs. C: 5.2 ± 4.1 (*p* > 0.05).	

Legend: AN: Patients with Anorexia Nervosa; BN: Patients with Bulimia Nervosa; C: Control Group; ED: Patients with Eating Disorders; SD: Standard Deviation; DMF: Decayed, Missing, Filled; DMFS: Decayed, Missing, Filled Surfaces; DMFT: Decayed, Missing, Filled Tooth; PAI: Periapical Index; d.f: Degree of Freedom

Only 5 studies identified a higher prevalence of caries among patients with eating disorders, while 5 did not find differences. Four studies have found only specific aspects related to higher caries prevalence within the ED group. Altshuler et al. 1990 reported a similar mean DMFS between patients with BN and control but observed a greater ‘decayed’ component in the BN group [22]. Similarly, Rytömaa et al. 1998 did not find a difference in DMFS and DS among BN patients but he found more pre-cavitation caries, approximal caries, and bucco-lingual caries in the BN group [14]. Conversely, Mascitti et al. 2019 found a higher mean DMFT in AN patients but no significant difference in the ‘decayed’ component [36]. Likewise, Ohrn et al. 1999 found no difference in ‘decayed’ data but observed significant disparities in DFS and DMFS. It is noteworthy that the difference in DS becomes apparent when considering the age range of 21–30 [41].

### Salivary function

A total of 19 studies conducted assessments of salivary flow rate, collectively involving 1,502 patients (Table 4). Among these studies, 9 included patients with bulimia nervosa, 2 with anorexia nervosa, and 8 studies encompassed a group that comprised various eating disorders concurrently. Of these, 14 studies collected and evaluated samples of whole saliva, 4 studies specifically assessed saliva from the parotid gland, and one study conducted separate evaluations of both whole saliva and parotid saliva production. Thirtheen studies reported a lower flow rate in patients with ED, while six studies found no differences. Lesar et al. 2022 did not observe differences between ED and C groups but did find significant differences between AN and BN [30]. Dynesen et al. 2008 identified statistically significant differences in unstimulated flow rate but not in paraffin-stimulated flow rate [31]. Johansson et al. 2015 and Rytömaa et al. 1998 did not find differences in stimulated and unstimulated flow rates but observed distinctions in terms of the proportions of patients with low unstimulated flow rates (< 0.1 and < 0.2 ml/min, respectively, in their studies) [14, 45].

**Table 4:** Main results salivary function

Author	Assessment Criteria	Salivary flow	Salivary pH
Aframian et al. 2010 [18]	Oral surface pH was measured from eight locations with a flat, glass electrode pH meter. Each set of measurements took approximately 40 s.	\	In all sites, mucosal pH levels were the lowest in the BN group except the posterior tongue location. Mean pH. BN 6.38 ± 0.45 vs. C 6.82 ± 0.33 (*p* = 0.02).
Altshuler et al. 1990 [22]	Salivary flow from Stensen’s duct was noted by using a pHydrion strip. Xerostomia was recorded upon complaint of dryness in the mouth and when no more than 5 ml of parrafin-stimulated saliva was produced in a 5-minute period.	Xerostomia was observed exclusively in 26 bulimic subjects (65%). A chi-square test demonstrated that the 20 bulimics (50%) who exhibited xerostomia were also likely to exhibit parotid dysfunction (chi-square = 14.43, *p* < 0.001). Six bulimics (15%) exhibited parotid dysfunction without xerostomia. Normal parotid function in the presence of xerostomia was observed in 2 bulimics (5%). Twelve bulimics (30%) exhibited normal parotid function and no xerostomia.	\
Blazer et al. 2008 [23]	Whole salivary collection under resting conditions for 5 min. Salivary flow rate (SFR). pH value. Questionnaire.	No significant difference in SFR. Questionnaire: Complaints of xerostomia were more prevalent among the BN patients (*p* < 0.003). 62% of C did not complaint of xerostomia, whereas 77% of the BN patients did. 31% of the BN patients complained of moderate to severe xerostomia, whereas the other 46% of the BN patients complained of mild xerostomia. Similarly, 47% of the BN patients complained of taste disturbances and/or burning sensorial disturbance in the oral cavity, whereas only 19% of C had similar complaints and the difference was significant (*p* = 0.016). No differences regarding the prevalence of the need for mouth rinsing or regarding difficulties in mastication, swallowing or communication.	The median salivary pH of the BN patients was significantly lower than that of the controls. Mean pH (range). BN: 6.58 ± 0.12 (5.5–7.6) vs. C: 6.88 ± 0.10 (5.2–7.6) (*p* < 0.05).
Chiba et al. 2019 [39]	Unstimulated salivary samples were collected. SFR.	The ED group exhibited lower SFR compared to the CN group. Salivary flow rate (mL/min). ED: 0.32 ± 0.13 vs. C: 0.49 ± 0.19 (*p* = 0.0001).	\
Dynesen et al. 2008 [31]	Unstimulated (UWS) and stimulated by paraffin whole saliva (SWS) was collected. Stimulated parotid saliva (SPS) was collected by a Lashley cup from the right parotid gland. Stimulated submandibular and sublingual saliva (SSS) was collected. Salivary flow rate (weighing). pH value. Questionnaire: Udvalg for Kliniske Undersogelser rating scale [65].	The UWS flow rate was significantly lower (*p* = 0.007) in the BN group. Flow rate (mL/min) mean ± SD. BN: 0.16 ± 0.14 vs. C: 0.29 ± 0.17 (*p* = 0.006). BN person with a daily intake of medication had a mean UWS flow of 0.08 ± 0.05 mL/min, whereas persons from the BN group with no daily intake of medication had a mean UWS flow rate of 0.23 ± 0.15 mL/min (*p* = 0.028). ED duration had a significant inverse effect on UWS flow rate (*p* = 0.019). The frequency of hyposalivation in BN (UWS flow rate < 0.1 mL/min33) was higher than in the C group. Nine BN persons suffered from hyposalivation, and 6 out of these 9 had an intake of medication. Only 1 person in the C group had such a low UWS flow rate. No differences between the BN group and the C concerning the SWS flow rate induced by chewing paraffin. However, the subgroup consuming medicine in the BN group had a significantly lower (*p* = 0.01) mean SWS flow rate (0.55 ± 0.33 mL/min) than the rest of the group. Questionnaire: Oral dryness was significantly more pronounced in the BN group (60%) than in the control (0%) (*p* = 0.003).	The pH values did not differ between the groups in any of the collected saliva samples.
Garrido-Martínez et al. 2019 [43]	Non-stimulated salivary flow measurements were made using the draining technique to determine the flow rate expressed as ml/min for 5 min. The results were classified as: normal salivary flow (> 0.3 ml/min), reduced (≤ 0.3 ml/min - ≥0.1 ml/min) and hyposialia (< 0.1 ml/ min). Salivary pH was evaluated as a quantitative variable, using pH Test Strips.	Mean salivary flow ± SD. ED: 0.23 ± 0.1 vs. C: 0.61 ± 0.27 (*p* < 0.001). Twelve patients in the ED (20.3%) obtained a non-stimulated flow of less than 0.1 ml/min, considered to represent hyposialia.	No statistical difference Mean pH ± SD. ED: 6.78 ± 0.52 vs. C: 6.75 ± 0.33 (*p* > 0.05).
Johansson et al. 2012 [33]	Unstimulated and paraffin-stimulated whole saliva were collected for periods of 15 and 5 min, respectively. SFR.	No difference in stimulated and unstimulated SFR. Unstimulated saliva < 0.1 ml min^− 1^). ED: 39% vs. C: 21% (*p* = 0.04). Stimulated saliva < 0.7 ml min^− 1^) ED: 11% vs. C: 6% (*p* > 0.05).	\
Johansson et al. 2015 [34]	Unstimulated whole saliva and paraffin-stimulated whole saliva were collected. Secretion rate. Questionnaire.	No difference in mean ± SD stimulated (ED: 0.22 ± 0.19 vs. C: 0.27 ± 0.21) and unstimulated salivary flow (ED: 0.64 ± 0.88; C: 0.66 ± 0.90). The proportion of subjects with unstimulated hyposalivation (a secretion rate of ≤ 0.1 ml/min) was significantly higher in the ED group compared with the control group (39% vs. 21%, respectively; *p* = 0.025). Questionnaire: Seventeen per cent of patients with EDs reported daily xerostomia compared with 6% of control subjects, whereas xerostomia once a month or more was reported by 52% and 30% of patients with ED and control subjects, respectively (*p* = 0.004).	\
Lesar et al. 2022 [30]	Whole unstimulated saliva samples were collected. Salivary flow, expressed in milliliters in the fifth and fifteenth minutes, was determined. Subjective sensation of saliva volume (decreased/normal/increased).	There is a significant difference in the volume of saliva secreted in the 5th (*p* = 0.007) and 15th minute (*p* = 0.028) between the AN and BN subgroups, whereas no significant difference was observed between the ED and C groups (*p* > 0.1). Median 5 min (ml) saliva volume, min-max. AN 0.8, 0-2.3 vs. BN 1.9, 1.2–2.8 vs. EDNOS 1.2, 0.5–3.3 vs. C 1.4, 0.1–8.5 (*p* = 0.005). Patients do not report differences in subjective perception of salivation volume.	\
Lourenço et al. 2018 [32]	Xerostomia was assessed based on patient’s complaints of dry mouth and difficulties in performing oral functions [31, 66]. The modified Schirmer’s test, performed with sterile paper strips, was used to evaluate the non-stimulated salivary flux (NSSF). Subjects with NSSF ≤ 25 mm, following 3 min of collection, were considered to have hyposalivation [66].	Xerostomia, hyposalivation and self-reported difficulties during oral function presented a relation with ED (*p* < 0.001). A similar trend was observed through data analysis of both vomit group and non-vomit group, where hyposalivation differed between the two groups (*p* = 0.02), whereas xerostomia did not (*p* = 0.21).	\
Milosevic and Dawson 1996 [15]	Stimulated SFR by chewing gum. pH value.	BN groups had significantly lower mean SFRs for both the initial 3-min and the overall 9-min compared with the C group (*p* < 0.01). There were no significant flow rate differences between the BN groups (ToothWear + and TW-) (*p* > 0.05). 3-min mean ± SD. BN TW+: 2.62 ± 0.74 vs. BN TW-: 2.02 ± 1.00 vs. C: 3.63 ± 0.97 (*p* < 0.01). 9-min mean ± SD BN TW+: 1.92 ± 0.57 vs. BN TW-: 1.45 ± 0.74 vs. C: 2.34 ± 0.68 (*p* < 0.01).	No differences in pH. BN TW+: 7.02 ± 0.21 vs. BN TW-: 7.07 ± 0.39 vs. C: 7.09 ± 0.18 (*p* > 0.05).
Ohrn et al. 1999 [41]	Unstimulated and paraffin stimulated. SFR. pH value. Buffer capacity.	Unstimulated SFR. <0.1 ml/min ED: 27% vs. C: 2% (*p* < 0.001). <0.2 ml/min ED: 50% vs. C: 35% (*p* < 0.001).	Stimulated saliva buffer capacity pH < 4.5. ED: 35% vs. C: 10% (*p* < 0.05).
Paszynska et al. 2006 [24]	Parotid saliva collected under unstimulated and stimulated conditions by a modified Lashley cap placed over Stensen’s duct under three different salivary flow conditions: after 15 min rest, physiologically stimulated using 3% citric acid applied to the tongue at 30 s interval and finally when stimulated by the mastication of wax tablets for 5 min. pH value.	The parotid SFR in BN subjects were significantly lower than C at rest and under stimulation. 40% of the subjects in group BN had unstimulated SFRs < 0.01 ml/min. Unstimulated SFR (ml/min). BN: 0.02 ± 0.01 vs. C: 0.08 ± 0.05 (*p* < 0.001). Stimulated 3% citric acid SFR (ml/min). BN: 0.2 ± 0.1 vs. C: 0.4 ± 0.2 (*p* < 0.001). Stimulated by mastication SFR (ml/min). BN: 0.08 ± 0.02 vs. C: 0.2 ± 0.1 (*p* < 0.001).	Statistically significant differences only in the unstimulated SFR. Unstimulated pH. BN: 7.2 ± 0.7 vs. C: 7.6 ± 0.5 (*p* < 0.05).
Paszynska et al. 2013 [19]	Parotid SFR was collected (by modified Lashley cap) under unstimulated and stimulated (3% citric acid and mastication) conditions.	BN had the lowest unstimulated and stimulated parotid SFR. Unstimulated parotid SFR (ml/min) BN: 0.02 ± 0.01 vs. C: 0.08 ± 0.05 (*p* ≤ 0.0001). Stimulated 3% citric acid SFR (ml/min). BN: 0.22 ± 0.11 vs. C: 0.45 ± 0.2 (*p* ≤ 0.001). Stimulated by mastication SFR (ml/min). BN: 0.08 ± 0.02 vs. C: 0.2 ± 0.1 (*p* ≤ 0.001).	\
Paszynska et al. 2014 [25]	Saliva was collected under both unstimulated conditions and 15 min later stimulated by chewing wax tablets for 5 min. pH value (fully automatic acid-base balance analyzer).	\	Unstimulated whole saliva pH was lower in AN group (6.6 ± 0.3) than in C group (6.8 ± 0.2); (*p* = 0.0001). After masticatory stimulation pH was 7.1 ± 0.1 in AN group and 7.2 ± 0.16 in C (*p* = 0.0011).
Paszynska et al. 2015 [26]	SFR. Saliva was collected under both unstimulated and stimulated conditions: at rest for 15 min and then stimulated by chewing wax tablets for 5 min.	Both stimulated and unstimulated SFR were significantly lower (50% for unstimulated and 24% for stimulated SFR) in the AN group than in the C group (*p* ≤ 0.001). In the AN group, 25% of the subjects had unstimulated SFR < 0.2 ml/min, while in 14%, the rate was < 0.1 ml/min. Under stimulated conditions, there were 1% subjects in the AN group with salivary flow < 0.8 ml/min (*p* ≤ 0.0005). No subject in the control group had unstimulated SFR < 0.2 ml/min and stimulated SFR < 0.8 ml/min. Unstimulated SFR. AN mean 0.27, median {0.30}, range 0.10–0.50, SD (0.12) vs. C: 0.54 {0.55}, 0.30–0.98, (0.18) (*p* = 0.0001) Stimulated SFR. AN: 1.20, {1.15}, 0.6–1.9, (0.34) vs. C:1.57 {1.50} 0.69–2.60 (0.46) (*p* = 0.0005).	\
Paszynska et al. 2017 [27]	Parotid unstimulated SFR collected by modified Lashley cap.	Parotid unstimulated SFR was significantly lower in the AN compared to C group. AN: 0.05 ± 0.03vs C: 0.09 ± 0.04 (*p* = 0.0039). 30% of AN subjects had unstimulated SFR at 0.02 ml/min, while no C subject had such low flow.	\
Philipp et al. 1991 [20]	pH value of the whole saliva was measured using a microglass electrode.	\	The pH value of saliva was reduced in all patients (*p* < 0.001). Bulimic patients had the lowest pH values. AN: 6.5 vs. BN: 6.4 vs. C: 7.
Riad et al. 1991 [17]	Parotid saliva was collected by Carlson-Crittenden cups. SFR for each gland was recorded under unstimulated condition and then stimulated by by applying 2 ml of a 5% citric acid solution to the tongue. The stimulated secretions were then collected for an additional 2 min.	BN patients had a reduced parotid resting SFR (*p* < 0.001). This was further decreased in the patients who developed sialadenosis. The stimulated SFR was reduced only in the sialadenosis group.	\
Rytomaa et al. 1998 [14]	Unstimulated and stimulated saliva samples were collected over 5-min periods. SFR.	No difference in the mean SFR between BN group and C (stimulated (1,9 vs. 19,6) and unstimulated (0,3 vs. 0,4)), but the number of subjects with low unstimulated SFR (< 0.2 ml/ min) was three times higher among BN than C (31% vs. 8%, *p* < 0.05). The feeling of dry mouth was three times commoner among BN patients than C (*p* < 0.001, BN 34% vs. 10%), and BN also had an increased tooth sensitivity to cold (54% vs. 10%) and touch (43 vs. 8%) (*p* < 0.001).	\
Schlueter et al. 2012 [28]	5 min of resting and 2 min of paraffin stimulated saliva was analysed. Additionally, from bulimic patients, 5 min of saliva was collected at home directly and 30 min after vomiting was investigated. SFR (weighing). pH value (ion-selective electrode).	No difference in flow rate was observed between the groups, both in the resting and stimulated conditions. Resting. BN 0.35 ± 0.18 vs. C 0.56 ± 0.25 (*p* > 0.05). Stimulated. BN 1.86 ± 1.02 vs. C 1.86 ± 0.86 (*p* > 0.05).	No differences in unstimulated pH. Stimulated pH in the BN without erosion group was significantly higher than in the C. BN no-erosion: 8.20 ± 0.67 vs. BN erosion: 7.89 ± 0.52 vs. C: 7.53 ± 0.32 (*p* < 0.001).
Touyz et al. 1993 [21]	SFR. pH value.	No significant difference in stimulated SFR was found. AN: 0.7 ± 0.4 vs. BN: 1.0 ± 0.6 vs. C: 0.92 ± 0.6 (*p* > 0.05).	Mean ED salivary pH was lower than C. AN: 7.1 ± 5 0.4 vs. BN: 7.1 ± 1.7 vs. C: 7.6 ± 0.3 (*p* < 0.001).

Legend: AN: Patients with Anorexia Nervosa; BN: Patients with Bulimia Nervosa; C: Control Group; TW: Tooth Wear; ED: Patients with Eating Disorders; UWS: Unstimulated Whole Salivary Flow; SD: Standard Deviation; SFR: Salivary Flow Rate; SWS: Stimulated Whole Saliva; SSS; Stimulated Submandibular and Sublingual Saliva; SPS: Stimulated Parotid Saliva; NSSF: Non-Stimulated Salivary Flux

### pH value

Eleven studies assessed the pH value (811 patients). Six evaluated BN patients, 1 AN, and 4 multiple EDs. Seven studies found a lower pH in patients with ED (1 AN, 3 BN, 3 MIX), 3 studies found no differences (2 BN, 1 MIX), while one study on BN patients found no differences in the unstimulated condition but reported a higher pH in stimulated saliva among BN patients who presented with dental erosion (Table 4).

### Periodontal & hygienic parameter

Out of the studies that assessed periodontal and hygiene parameters, a total of 11 studies were included in this analysis (Table 5). Among these, two studies focused specifically on patients with AN, another two on individuals with BN, and the remaining seven encompassed groups with multiple diagnoses of EDs. In total, these studies involved 1,190 patients. A variety of heterogeneous clinical indices were employed for assessment in these studies. Six studies found comparable or lower probing depths between the ED group and the control group, and none of the studies reported a higher prevalence of periodontitis or increased probing depths in the ED group. The diagnostic criteria used in Lourenço et al.‘s study, which identified patients with gingival recession or probing depth greater than 3 mm as cases of periodontitis, are no longer consistent with the current classification and may lead to incorrect diagnoses [32]. Consequently, those findings related to periodontitis were excluded from the analysis.

**Table 5:** Main results periodontal & hygienic parameter

Author	Assessment criteria	Main results
Altshuler et al. 1990 [22]	Gingival index [67]. Periodontal index [68] Plaque index [69].	No significant differences in gingival index, periodontal index and plaque index (*p* > 0.05).
Chiba et al. 2019 [39]	Community Periodontal Index (CPI). The highest code was recorded per sextant [70].	The mean of the CPI was higher in the ED group than in C group, showing that patients with AN and BN had worse periodontal conditions compared to the C group (*p* < 0.05), with no significant differences observed in periodontal pockets of 4–5 mm. Mean number of sextants ± SD according to periodontal status evaluated by CPI: Healty gingiva. ED: 2.07 ± 1.84 vs. C: 5.53 ± 0.73 (*p* < 0.0001). Bleeding on probing. ED: 1.87 ± 1.48 vs. C 0.33 ± 0.61 (*p* < 0.0001).
Garrido-Martínez et al. 2019 [43]	The Ramfjord Periodontal Index [71].	The periodontal status was similar in both groups. Mean Ramfjord Periodontal Index ± SD. ED: 1.55 ± 0.94 vs. C: 1.49 ± 0.93 (*p* > 0.05).
Johansson et al. 2012 [33]	Visible plaque index (VPI) and gingival bleeding index (GBI) were recorded for each tooth on the buccal, mesio-buccal, and lingual surfaces [72].	VPI and GBI were significantly lower in ED group. Median VPI % (range). ED: 7.1 (0–51) vs. C: 11.3 (0–39) (*p* = 0.01). Median GBI % (range). ED: 1.0 (0–38) vs. C: 7.1 (0–30) (*p* = 0.001).
Lourenço et al. 2018 [32]	Participants presenting visual signs of generalized gingival inflammation, with bleeding and pain after probing, were considered to have gingivitis [73].	No significant differences were found between groups regarding gingivitis (*p* > 0.05).
Mascitti et al. 2019 [36]	Plaque index, periodontal probing depth, clinical attachment level, Periodontal Screening and Recording Index and presence of bleeding on probing [74].	No significant difference in gingivitis (23 AN patients vs. 16 C) and periodontitis (2 AN vs. 3 C) (*p* = 0.6378).
Pallier et al. 2019 [42]	Full-mouth periodontal examination, 6 sites per tooth. Plaque control was evaluated using a dichotomized plaque index [75], and gingival inflammation using bleeding on probing. Probing depth (PD), and gingival recession (REC) were measured in millimeters. Clinical attachment level was calculated as the sum of PD and REC.	Mean percentages of sites with dental plaque and bleeding on probing were higher among ED participants than among controls (71.5 ± 26.8 and 30.2 ± 26.3 versus 53.0 ± 20.4 and 21.8 ± 18.7, *p* < 0.01 and *p* = 0.03 respectively). ED patients presented more than 2% of sites with gingival recession ≥ 3 mm, while none had gingival recession exceeding 2 mm among controls. % of sites with REC > 2 mm ± SD. ED: 2.3 ± 4.1 vs. C: 0.0 ± 0.1 (*p* < 0.01). % of sites with PPD > 3 mm ± SD. ED: 0.5 ± 1.7 vs. C: 3.1 ± 7.3 (*p* < 0.01).
Paszynska et al. 2022 [13]	Plaque Control Record index (PCR) [75] and Bleeding on Probing index (BOP) [76] were measured at six points per tooth.	Mean percentages of sites with dental plaque and BoP were significantly higher among AN patients than in controls (43.8 ± 23.4 and 20.0 ± 20.1 vs. 13.7 ± 15.4 and 3.9 ± 8.1, *p* < 0.001, twice respectively) ED patients with purging habits have more plaque and gingival inflammation than those without purging (*p* < 0.005).
Philipp et al. 1991 [20]	Approximal plaque index (API) [77]. Sulcus bleeding index (SBI) [78]. Loss of attachment of six representative teeth was measured [79].	ED patients had reduced API-values and significantly reduced gingival inflammation API. AN: 24% ± 15 vs. BN: 26% ±20 vs. C: 52% ± 28 (*p* < 0.0011). SBI. AN: 14% ± 13.5 vs. BN: 12.5% ± 14.5 vs. C: 44% ± 25.9. (*p* < 0.0011). No patients experience clinical attachment loss.
Rytomaa et al. 1998 [14]	CPTIN [70]. Oral hygiene plaque index [72]. Gingival index [72].	No difference between bulimics and controls was seen in oral hygiene habits and periodontal status (DNS).
Touyz et al. 1993 [21]	The CPITN [70] was assessed for all sextants of teeth in each subject. The Plaque Index [69] was recorded for both facial and lingual surfaces of six representative teeth.	Controls had significantly more surfaces with a plaque score of 0 (i.e., no plaque) than ED patients. AN patients had significantly greater number of sites with gingival recession of 1–3 mm than BN and C (10.2, 3.0 and 2.0, respectively; *p* < 0.001). They also had a greater number of sites that bled on probing (16.9, 9.4 and 6.5, respectively; *p* < 0.001). No differences in pocket depth ≥ 4 mm. When CPITN scores were considered, AN patients had lower mean numbers of healthy sextants and higher mean numbers of sextants with bleeding compared with the BN, and both had significantly less healthy sextants than the control groups. These differences were statistically significant for scores of 0 and 1 (DNS).

Legend: AN: Patients with Anorexia Nervosa; BN: Patients with Bulimia Nervosa; C: Control Group; ED: Patients with Eating Disorders; SD: Standard Deviation; DNS: Data Not Shown; CPI: Community Periodontal Index; VPI: Visible Plaque Index; GBI: Gingival Bleeding Index; PD: Probing Depth; REC: Gingival Recession; PCR: Plaque Control Record Index; BOP: Bleeding on Probing Index; API: Approximal Plaque Index; SBI: Sulcus Bleeding Index; CPTIN: Community Periodontal Index of Treatment Needs

Four studies reported higher levels of gingival bleeding on probing in individuals with ED while four studies found similar levels, and two studies observed lower levels in the ED group. Regarding plaque indices, three studies identified a greater quantity of plaque in ED patients, two studies found no difference, and two studies reported less plaque.

Two studies assessed the prevalence of gingival recession, and both reported a higher occurrence in patients with ED. An internal comparison within the ED group conducted by Touyz et al. 1993 revealed that anorexic patients had more sites with recession compared to bulimic individuals and the control group [21].

### Oral mucosal tissues

Table 6 displays the seven included studies for a total of 701 patients (1 study on patients with AN and 6 studies with multiple ED diagnoses). The majority of studies have reported a notable frequency of soft tissue pathologies in patients with ED. Garrido-Martínez et al. 2019 found a soft tissue affectation prevalence of 98% and 43.5% in ED and control groups, respectively, while Panico et al. 2018 reported 94% and 18.5% [16, 43]. The most common oral pathologies include angular cheilitis/exfoliative cheilitis, labial erythema, and burning tongue/burning mouth. In populations of similar age, the study by Johansson et al. in 2012 identified cases of parotid gland enlargement in the ED group (1 out of 4 patient with AN, 4/8 BN, 12/32 EDNOS vs. 0/54 in the control group), while Panico et al. 2018 did not find any [16, 33].

**Table 6:** Main results oral mucosal tissues

Author	Assessment criteria	Main results
Garrido-Martínez et al. 2019 [43]	Clinical assessment.	ED patients presented more soft tissue lesions (98%) than C (42.5%) (*p* < 0.001). There was found statistically significant differences for dry lip, angular cheilitis, erythema, ulcerations (*p* < 0,001) and saburral tongue (*p* < 0.05).
Johansson et al. 2012 [33]	Clinical assessment. Questionnaire.	Clinical examination: Parotid gland enlargement was found in 31% (*n* = 17) of patients with EDs (one with AN, four with BN, and 12 with EDNOS) but in none of the controls (*p* = 0.001). Patients with esophagitis were 2/14, 2/8, 2/32 for the AN, BN, and EDNOS groups, respectively, with no instances observed in the C group. Questionnaire: Signs of dry and/or cracked lips, mouth dryness, burning tongue or parotid gland swelling, were significantly more common in ED patients than in controls (*p* < 0.001). Swelling in front of the ear (parotid enlargement) was only reported in the ED group (four patients).
Lourenço et al. 2018 [32]	Clinical assessment.	Angular cheilitis and burning mouth feeling were found to be significantly more common in EDG. Vomiting appears to have no impact on stomatodynia, while it may have an effect on angular cheilitis. Exfoliative cheilitis, fissured tongue, and lichen planus did not differ significantly between groups. No cases of actinic cheilitis, oral candidiasis, or soft palate lesions were identified in either group.
Panico et al. 2018 [16]	Clinical assessment by two previously calibrated odontologists. The final diagnosis oral mucosal lesions were reached through consensus of the two examiners; in cases of disagreement, the diagnosis was defined by a third part.	In the ED group 94% (*n* = 61) showed oral lesions (a total of 112 lesions), while control group had 18.5% (*n* = 12, 15 lesions). Common oral lesions more prevalent in ED patients (*p* < 0.03) were labial erythema (43% ED vs. 0% C), exfoliative cheilitis (43% vs. 10.7%), orange-yellow palate (35% vs. 1,5%), hemorrhagic lesions (26% vs. 4.6%), lip-cheek biting (18% vs. 6.1%) and non-specific oral atrophies (7.7% vs. 0%). Considering the most common oral lesions in the study group, there was a statistically significant difference with control (OR 50.8, CI 95%, 15.8-162.9, *p* < 0.0001). Only labial erythema differs among the various subgroups of ED (more common in BN compared to AN and EDNOS; *p* < 0.0098). Only labial erythema was associated with a higher frequency of vomiting per day (3 vomits/day vs. 1.89; *p* = 0.03). Considering all the oral lesions together, they were related only to purging habits (OR 6, CI 95%, 1.06–34.12, *p* = 0.0414). However, considering the oral lesions independently, only labial erythema displayed a statistically significant association with self-induced vomiting (OR 4, CI 95%, 1.08–14.77, *p* = 0.0396) and diuretic/laxative use (OR 7.89, CI 95%, 2.18–28.56, *p* = 0.0012). No case of major salivary gland swelling was reported.
Paszynska et al. 2014 [25]	Clinical assessment.	ED clinical assessment revealed: exfoliate cheilitis (13 subjects; 41.9%), pallor of the oral mucosa and skin (9; 29.0%), atrophic glossitis (8; 25.8%), white coating of the tongue (8; 25.8%), linea alba (6; 19,3%), erythematous spots on the palate (4; 12.9%), morsicatio buccarum (4; 12.9%), angular cheilitis (4; 12.9%) and ulcers of a traumatic etiology (4; 12.9%). C group assessment: white coated tongue (10 subjects; 25.0%), linea alba (9; 22.5%) and geographic tongue (1; 2.5%). The subjective symptoms experienced by patients with AN included a burning sensation of the oral mucosa in 4 subjects (12.9%) compared to 0 in the C group.
Philipp et al. 1991 [20]	Clinical assessment. Swelling and inflammation of the parotid glands were divided into three grades. Grade 1: inconspicuous; grade 2: swelling without inflammation; and grade 3: swelling with inflammatory changes.	Bilateral, mostly painless facial swelling was observed in 27 of 41 bulimic patients. The intensity of parotid enlargement correlated with the severity of enamel erosions. There was a significant correlation between changes of the parotid glands and the number of decalcified surfaces of teeth in bulimic patients with an anorectic prephase (*p* < 0.05).
Touyz 1993 [21]	Clinical assessment.	No AN or C subject had any sign of parotid gland enlargement whereas 3 BN patients did.

Legend: AN: Patients with Anorexia Nervosa; BN: Patients with Bulimia Nervosa; C: Control Group; EDNOS: Patients with Eating Disorder Not Otherwise Specified; ED: Patients with Eating Disorders; SD: Standard Deviation; OR: Odds Ratio; CI: Confidence Interval

### Other

Other oro-dental outcomes assessed in a smaller number of studies included hypersensitivity (*n* = 2), temporomandibular disorders (*n* = 2), parafunctional habits (*n* = 1), and malocclusion (*n* = 1). Both studies on hypersensitivity reported a higher prevalence among individuals with EDs, whether self-reported or induced by air or explorer stimuli (Table 7). Similarly, malocclusion and various aspects related to temporomandibular disorders appeared to be more prevalent in individuals with ED, who also seemed to report a higher occurrence of muscle disorders, facial pain, earache, headache, and burning sensations in the mouth.

**Table 7:** Main results “other” oro-dental outcomes

Author	Assesment criteria	Main results
Altshuler et al. 1990 [22]	Dentin hypersensitivity was recorded per tooth surface (facial, lingual, or occlusal tooth surface) when the patient reported a history of symptomatology and a response was elicited from a 5-second blast of compressed air and/or contact with a dental caries explorer.	There is increased sensitivity in patients with ED (*p* = 0.01), primarily localized in the anterior region (*p* < 0.01) rather than the posterior (*p* > 0.05). 27 bulimic subjects (68%) reported a history of hypersensitivity and had a reaction to the dentin hypersensitivity test compared to 13 C subjects (33%).
Chiba et al. 2022 [40]	Dental Aesthetic Index (DAI) was used to evaluate the prevalence of malocclusions.	Severe or disabling malocclusion was significantly higher in the ED group than in the C. ED: 22/30 (73.3%) vs. C: 4/30 (13.3%) (*p* < 0.004). ED group showed a higher proportion of patients (*p* < 0.05) with upper teeth loss, lower teeth loss, spacing in the region of incisors, anterior maxillary misalignment, and anterior mandibular misalignment in relation to C group. ED group showed a significantly higher (*p* < 0.05) DAI score compared to the C. Mean DAI score ± SD. ED: 38.33 ± 10.65 vs. C: 21.33 ± 8.58 (*p* < 0.0001).
Johansson et al. 2010 [29]	Temporomandibular disorder: Clinical examination by a TMD specialist. Diagnosis was made according to the epidemiological variable TMD-S subsequently termed TMD pain [80]. Questionnaire: Helkimo’s Anamnestic and Clinical Dysfunction Indices [81].	TMD pain was diagnosed in 28 ED patients (48%) and 11 controls (20%) (*p* = 0.04). Maximum opening capacity of the mouth was significantly lower among ED patients (mean = 52 mm, SD = 5.6) compared to controls (mean = 54 mm, SD = 5.4) (*p* = 0.043). Higher TMD signs and symptoms were found in ED patients compared to C (Anamnestic Index, *p* = 0.05; Dysfunction Index, *p* = 0.009). No significant differences regarding pain on mandibular movement, difficulties in wide opening, clicking or grating sounds from TMJ’s, locking of TMJ, tense in the jaws in the morning, bruxism, chewing problems, subjective symptoms and clinical signs of TMD were found.
Lourenço et al. 2018 [32]	Dentin hypersensitivity was based on patients’ self-report to cold, sweet, or acidic stimuli.	Self-reported dentin hypersensitivity was found to be significantly higher for both vomit and non-vomit group, as compared to controls (*p* < 0.01).
Manevski et al. 2020 [35]	Questionnaire: parafunctional habits (grinding and clenching teeth, nibbling of foreign objects).	No differences in grinding and clenching or nibbling of foreign objects (*p* > 0.7).
Pereira de Souza et al. 2018 [37]	Orofacial pain. Temporomandibular Disorder. Validated Portuguese version of the Research Diagnostic Criteria for TMD questionnaire.	Complaints of pain in ED patients were more prevalent in individuals with ED (*p* < 0.004). Facial pain ED: 54.7% (*n* = 23) vs. C: 9.1% (*n* = 2); earache 50% (*n* = 21) vs. 4.5% (*n* = 1); sore throat 23.8% (*n* = 10) vs. 13.6% (*n* = 3); headache 52.3% (*n* = 22) vs. 4.5% (*n* = 1); burning in the mouth 26.2% (*n* = 11) vs. 0%; pain in other regions of the body 45.23% (*n* = 19) vs. 4.5% (*n* = 1). In the diagnosis of TMD, the ED groups exhibited statistical differences regarding muscle disorders (*p* = 0.010) and other joint changes on the left side (*p* = 0.006).

Legend: AN: Patients with Anorexia Nervosa; BN: Patients with Bulimia Nervosa; C: Control Group; TMD: Temporomandibular Disorders; TMJ: Temporomandibular Joint; DAI: Dental Aesthetic Index; ED: Patients with Eating Disorders; SD: Standard Deviation

## Discussion

Our systematic review highlights the need for more validated tools in the dental field for the effective management of ED-related oral conditions. It points out the prevalent dental erosion in patients with anorexia nervosa, bulimia nervosa, and EDNOS, and indicates a possible association between anorexia nervosa and higher tooth decay rates. The study underscores the importance of enhancing dental education regarding EDs, calls for more research into these correlations, and stresses the necessity for sensitive patient communication and holistic care approaches.

Infact, despite being formally trained in eating disorders, surveys among dentists and dental hygienists reveal a prevailing lack of familiarity in managing patients with EDs, along with difficulty in communicating suspicions about the disorder to patients or relatives [45]. This inadequacy might impact the limited referrals for medical treatment [46].

A recent scoping review highlighted the continued importance of ongoing research and updates in dental education regarding EDs [47]. The review found no recent evidence on this topic and reported that oral health practitioners generally lack sufficient knowledge of eating disorders and have limited clinical experience in this area. It emphasized that knowledge of oral signs is a critical factor that increases the likelihood of evaluation, referral, and case management.

Equally vital is the dissemination of information to medical practitioners regarding oro-dental manifestations, as currently, patients receiving treatment for EDs often fail to receive appropriate oral health care [48].

It is noteworthy that despite binge eating disorder (BED) being the most prevalent eating disorder [49], none of the studies included in the systematic review seemed to specifically address this issue. This could be due to BED being recognized as a distinct ED relatively recently, so there may be deficiencies in awareness and research on this specific topic. It is also important to consider the impact of weight stigma, which affects the physical and mental health of patients with obesity. This stigma could potentially serve as a barrier for healthcare professionals in recognizing and diagnosing obesity-related conditions. Additionally, patients may face challenges in explaining their difficulties due to weight-related stigma, further complicating their access to appropriate care and support [50].

The synthesis of extensive data from a wide spectrum of studies, including a considerable time span and the incorporation of newly eligible articles, characterizes this systematic review. Additionally, its identification of underexplored areas hints at significant opportunities for future research in this domain.

Erosion is a significant manifestation that has transversally involved patients with AN, BN and EDNOS. In all included studies, dental erosion consistently emerged as the predominant feature of patients with EDs, differing from the control group in terms of patient-level prevalence, tooth-level prevalence, extent, severity and location. Several studies established a direct relationship between vomiting episodes and/or purging behaviors and the occurrence of dental erosion [22, 32, 35, 43]. Alongside vomiting and compensatory behaviors, some harmful habits typical of individuals with EDs, such as frequent consumption of carbonated beverages and aggressive tooth brushing immediately after vomiting, might contribute to the onset and progression of dental hard tissue loss. Overall, it is plausible that various wear mechanisms interact, with the most significant interaction arising from the combination of mechanical abrasion and chemical erosion [51].

The requisite factors for the development of carious pathology are different. Tooth decay is an infectious disease that affects the calcified tissue of the tooth and causes the dissolution of the organic component and the demineralization of the inorganic portion. It is caused by the deposition of bacterial biofilm on the surface of the tooth and is favored by the frequent consumption of fermentable carbohydrates. Some oral microorganisms such as *Streptococcus mutans* metabolize fermentable carbohydrates and produce lactic acid, which lowers oral pH to a level where enamel and dentin minerals dissolve easily [52]. The marked heterogeneity present in the caries studies in this review it does not allow us to draw definitive conclusions; however, it should be noted that when analyzing the results based on EDs diagnosis (Table 8), the two studies involving individuals with AN both found a higher prevalence of the DMFT score [13, 36]. Furthermore, other studies with mixed diagnoses but with a notable presence of individuals with AN have shown a higher prevalence of caries [32, 38, 42]. Therefore we could hypothesize, albeit with absolute caution, that among the various eating disorders the only one that could be associated with the presence of tooth decay is anorexia nervosa.

Table 8 presents a summary of the results, specifically highlighting the number of studies that identified an association between ED and the oral outcome in relation to the number of studies investigating this aspect.

**Table 8:** A concise summary of the principal findings, indicating the proportion of studies demonstrating a connection between eating disorders and oral outcomes compared to the total number of studies exploring this aspect

Eating Disorder	Oro-dental outcomes				Oral mucosal tissues
	Erosion	Decay	Salivary flow rate	Salivary pH	
AN	2/2	2/2*	2/2	1/1	0/1
BN	5/5	1/4**	6/9**	4/6**	0/0
MIX	7/7	4/8*	5/8	3/4	4/6**
Total	14/14	7/14	13/19	8/11	4/7

AN: Anorexia Nervosa; BN: Bulimia Nervosa; MIX: Multiple Eating Disorder Diagnoses; *in one study, not all measured outcomes differed between the two groups; **in two studies, not all measured outcomes differed between the two groups

This connection could be due to factors such as dietary preferences, infrequent meals leading to extended acidic exposure for teeth, reduced salivary flow, which is crucial for neutralizing oral acids, variations in oral hygiene practices due to psychological stress impacting oral care, and nutritional deficiencies weakening teeth. It’s important to note that these are speculative associations based on behaviors commonly observed in individuals with anorexia nervosa. Further research is necessary to establish a definitive link, as this systematic review serves to highlight potential areas for future investigation rather than providing conclusive evidence.

Over 65% of studies observed reduced saliva flow. Variations in collection times, methodologies, and often unverified parameters such as medications, hormonal status, vomiting, nutritional deficiencies, and hydration complicate comparisons. Nevertheless, 5 out of 6 studies investigating patient complaints of xerostomia/oral dryness revealed statistically significant differences, affirming the perception of reduced salivation among the ED patients. Additionally, studies assessing minimal saliva quantity (0.1–0.2 ml/min) found a significantly higher proportion of patients with reduced saliva in the EDs group.

The heterogeneity among studies in assessing pH poses challenges in synthesizing existing evidence. Even when differences were observed between EDs and control groups, the closely aligned mean scores limit their clinical utility and relevance for the specific objectives of this systematic review. A more comprehensive approach might involve continuous 24-hour pH monitoring, allowing for a thorough assessment of mean pH, pH fluctuations, duration of acidic pH exposure, number of pH peaks, and salivary buffer capacity efficiency, despite the complexity of such examinations.

Recent articles have brought to light new evidence regarding oral soft tissue characteristics. Correlations have been identified between EDs and various oral manifestations such as dry lips, angular cheilitis, erythema of the palate and lips, palatal ulcers, coated tongue, yellow-orange palate, and more. These manifestations could relate to vomiting episodes (resulting in dehydration) or the use of diuretics and laxatives, although other contributing factors may also be involved. Eating disorders are commonly linked to psychological disturbances, including obsessive-compulsive behaviors or self-injurious behaviors such as cutting, burning of the skin, reopening of wounds, and other forms of self-harm [53]. Morsicatio buccarum, repeated biting of the cheeks or lips, coupled with hemorrhagic lesions, palatal and pharyngeal lesions (erythema and ulcers), might be considered indicative of EDs [54]. Obsessive-compulsive behaviors may lead to intense and frequent tooth brushing, which, on one hand, could explain the variability in plaque presence and gingivitis and, on the other hand, contribute to dental erosion and the development of gingival recessions [55].

The less explored oro-dental aspects in literature, categorized here as “other aspects,” could provide a new avenue for research in this field. Particularly, examining potential links between EDs, temporomandibular disorders and malocclusion holds significant interest, providing valuable insights into the potential impact of EDs on the structure and function of the stomatognathic system.

In assessing oro-dental manifestations, it is important to consider that certain alterations may require different durations to manifest. It can be hypothesized that alterations affecting soft tissues might act as more immediate indicators, potentially displaying quicker changes over time, as they could reflect not only for local changes but also for signaling systemic dysfunctions or alterations and pathologies belonging to different domains. Conversely, manifestations involving hard tissues might require a longer onset period and, once present, exhibit a worsening nature. The role of the oral health practitioners towards EDs patients could also be expressed in a rational and evidence-based use of active compounds towards the tooth mineral component. This emerging dental aspect is still under-investigated for patients with EDs and represents a crucial point for future investigations. Patient-reported symptoms such as dysgeusia, xerostomia and oral burning sensation may behave differently, potentially stemming from psychogenic elements and expressing somatization of underlying disorders [56].

Encouraging longitudinal studies that analyze diverse oro-dental aspects over time in young patients with EDs would be beneficial. Such research could elucidate potential causal connections and comprehend the sequential/chronological manifestation of different outcomes. Timely addressing of these manifestations is pivotal for prognosis, affecting dietary habits, function, self-image, and consequently, self-esteem [57].

Given the dental team’s primary role in aiding patients with eating disorders and the critical importance of timely treatment by mental health and medical experts, sensitive communication post-identification of specific oral manifestations becomes strategic [58].

### Limitations

This systematic review has inherent limitations. Overall, the studies’ quality was relatively modest, potentially affecting result precision due to methodological limitations. Wide ranges in age across studies, although mean ages were relatively similar, and patient recruitment from diverse populations, including hospitalized patients with potentially severe EDs, may both influence oro-dental manifestations. Furthermore, the role of pharmacotherapy as a variable, inconsistently verified and controlled, may impact oro-dental outcomes.

Predominantly female subjects from European regions were included in this study, which, while limiting generalizability to male individuals or non-EU populations, aligns with the prevalent statistics of eating disorders. This demographic focus is consistent with the higher incidence of eating disorders observed among females, as indicated by current prevalence data. However, it’s important to acknowledge this as a limitation in terms of the broader applicability of our findings to diverse populations and genders. While aligned with the review’s objective, heterogeneous study groups with the co-presence of different ED diagnoses may not have highlighted specific characteristics. Stratification of these groups in the statistical analyses of the respective studies could have allowed for a more specific evaluation of each ED’s characteristics. Variation in diagnostic criteria and DSM versions might have complicated identifying associations between different EDs and their oral implications, as did outcome measurement heterogeneity, hindering inter-study comparisons. It is conceivable that some studies [19, 24–26, 33, 34] may have been conducted on overlapping populations or on populations that are highly similar, although this was not explicitly reported. Lastly, nearly all studies lack examiner blinding, introducing potential bias due to knowledge of patient’s diagnosis.

## Conclusion

This systematic review comprehensively assessed the relationship between feeding and eating disorders (EDs) and their impact on oro-dental health, meticulously identifying, evaluating, and synthesizing findings from the existing body of scientific research. Our analysis of the collated data has underscored that certain oro-dental manifestations show a notable and consistent correlation across various studies, suggesting a robust association with EDs. These include conditions such as dental erosion, reduced salivary flow, and specific oral mucosal changes. However, it has also become evident that other oro-dental outcomes, particularly those relating to dental caries, pH value variations, and periodontal health, present a more complex picture and thus warrant further in-depth investigation. The findings of this review highlight the multifaceted nature of the impact of EDs on oral health and underscore the need for continued research to fully understand these associations and inform more effective clinical practices.

### Electronic supplementary material

Below is the link to the electronic supplementary material.

Supplementary Material 1

## Data Availability

No datasets were generated or analysed during the current study.

## Abbreviations

EDs: Eating Disorders
AN: Anorexia Nervosa
BN: Bulimia Nervosa
DMFS: Decayed, Missing, Filled Surfaces
DMFT: Decayed, Missing, Filled Tooth
EDNOS: Eating Disorder Not Otherwise Specified
BEWE: Basic Erosive Wear Examination
MIX: Multiple Eating Disorder Diagnoses
BED: Binge Eating Disorder
